# Neuroanatomical and Functional Correlates of Cognitive and Affective Empathy in Young Healthy Adults

**DOI:** 10.3389/fnbeh.2019.00085

**Published:** 2019-05-01

**Authors:** Carme Uribe, Arnau Puig-Davi, Alexandra Abos, Hugo C. Baggio, Carme Junque, Barbara Segura

**Affiliations:** ^1^Medical Psychology Unit, Department of Medicine, Institute of Neuroscience, University of Barcelona, Barcelona, Spain; ^2^Centro de Investigación Biomédica en Red sobre Enfermedades Neurodegenerativas, Hospital Clínic de Barcelona, Barcelona, Spain; ^3^Institut d’Investigacions Biomèdiques August Pi i Sunyer, Barcelona, Spain

**Keywords:** cognitive empathy, affective empathy, healthy subjects, cortical thickness, fMRI, resting-state connectivity, young adults, orbitofrontal cortex

## Abstract

Neural substrates of empathy are mainly investigated through task-related functional MRI. However, the functional neural mechanisms at rest underlying the empathic response have been poorly studied. We aimed to investigate neuroanatomical and functional substrates of cognitive and affective empathy. The self-reported empathy questionnaire Cognitive and Affective Empathy Test (TECA), T1 and T2^∗^-weighted 3-Tesla MRI were obtained from 22 healthy young females (mean age: 19.6 ± 2.4) and 20 males (mean age: 22.5 ± 4.4). Groups of low and high empathy were established for each scale. FreeSurfer v6.0 was used to estimate cortical thickness and to automatically segment the subcortical structures. FSL v5.0.10 was used to compare resting-state connectivity differences between empathy groups in six defined regions: the orbitofrontal, cingulate, and insular cortices, and the amygdala, hippocampus, and thalamus using a non-parametric permutation approach. The high empathy group in the Perspective Taking subscale (cognitive empathy) had greater thickness in the left orbitofrontal and ventrolateral frontal cortices, bilateral anterior cingulate, superior frontal, and occipital regions. Within the affective empathy scales, subjects with high Empathic Distress had higher thalamic volumes than the low-empathy group. Regarding resting-state connectivity analyses, low-empathy individuals in the Empathic Happiness scale had increased connectivity between the orbitofrontal cortex and the anterior cingulate when compared with the high-empathy group. In conclusion, from a structural point of view, there is a clear dissociation between the brain correlates of affective and cognitive factors of empathy. Neocortical correlates were found for the cognitive empathy dimension, whereas affective empathy is related to lower volumes in subcortical structures. Functionally, affective empathy is linked to connectivity between the orbital and cingulate cortices.

## Introduction

Empathy is the ability to understand the thoughts and feelings of others, and to respond to these feelings in an appropriate way. The construct of empathy can be divided into cognitive and affective dimensions (Walter, 2012; Gonzalez-Liencres et al., 2013; Dvash and Shamay-Tsoory, 2014), although the literature on this subject is not always clear due to the multidimensionality of the concept. Cognitive empathy-related processes such as the Perspective Taking dimension “occur through interactions between limbic and cognitive structures” (Decety et al., 2012). Indeed, literature on perception of others in distress or pain have linked somatosensory information with limbic affective and motivational components (Lamm et al., 2011; Decety et al., 2012).

In the past decades, there has been increasing interest in studying the neural basis of empathy with the emergence of magnetic resonance imaging (MRI) techniques in social neuroscience. Recent functional MRI (fMRI) studies have reported limbic structures such as the amygdala, the anterior insula, and the anterior cingulate cortex to be part of the neural bases of affective empathy (Lamm et al., 2011; Bernhardt and Singer, 2012; Gonzalez-Liencres et al., 2013). The prefrontal cortex, including dorsolateral, ventromedial, and orbitofrontal regions, would in turn be related to cognitive empathy (Van Overwalle and Baetens, 2009; Bernhardt and Singer, 2012). A meta-analysis carried out by Fan et al. (2011) including 40 studies concluded that the left anterior insula is recruited for both affective and cognitive empathy. By contrast, the right anterior insula and the right inferior frontal gyrus seem to be more related to affective-perceptual empathy while the left anterior cingulate cortex is involved in the process of cognitive empathy (Fan et al., 2011). In many of the functional MRI studies, empathy has been assessed as a state in performing a task-based MRI design rather than a trait (Lamm et al., 2007; Harvey et al., 2013; Braadbaart et al., 2014; Moore et al., 2015). Lately, few studies have investigated the functional connectivity of the brain at rest linked to the ability to empathize as an intrinsic feature (Takeuchi et al., 2014, 2018; Bilevicius et al., 2018) measured by questionnaires.

Similarly, there are few published structural MRI studies addressing the neuroanatomical substrate of empathic ability measured by questionnaires. The majority of these studies used regions selected *a priori* and a voxel-based morphometry (VBM) approach. Using VBM, Banissy et al. (2012) found negative correlations between scores of affective empathy and gray matter (GM) volumes in the left precuneus, inferior frontal gyrus, anterior cingulate cortex, somatosensory cortex, and the insula. By contrast, employing GM density measures, Eres et al. (2015) reported positive correlations in the insula. Cognitive empathy has been related to cingulate, dorsolateral, and dorsomedial prefrontal cortices (Banissy et al., 2012; Eres et al., 2015). Considering both affective and cognitive empathy, Goerlich-Dobre et al. (2015) described a positive correlation between GM volumes in the left amygdala, bilateral thalamus, and the left parahippocampal gyrus. In line with the findings on fMRI by Fan et al. (2011), the left anterior insula would also be a neuroanatomical substrate for global empathy (Mutschler et al., 2013). To date, only one previous study investigated the correlation between empathy scores and whole-brain cortical thickness (Valk et al., 2016). The authors found a positive correlation between empathy scores and cortical thickness in left inferior frontal, opercular, and insular gyri.

To the best of our knowledge, no previous studies have investigated structural and functional dissociations of affective and cognitive empathy in the same sample of subjects. The aim of the present study was to investigate the structural and functional substrates of empathy in the same sample of healthy young persons. We were interested specially in differences within regions of the limbic system. We hypothesized that cognitive and affective empathy would present distinct regional cortical thickness patterns in neocortical regions and subcortical volumetric differences. Similarly, individuals would differentiate in their functional brain connectivity depending on empathy levels (low empathy and high empathy groups).

## Materials and Methods

### Participants and Instruments

Fifty-six volunteers were recruited from advertising the study between students of the first course of the Nursing Bachelor of the University of Barcelona, Campus Clinic. These students were also invited to recruit friends or relatives of similar age and education. The inclusion criterion was that individuals would be between 18 and 35-years old. The exclusion criteria were: (1) presence of neurological or psychiatric disorders, (2) MRI incompatibilities such as metal implants that could not be extracted, (3) claustrophobia, (4) meeting DSM-IV criteria for substance abuse or dependence within the past year, and (5) current use of psychoactive medication.

Fourteen subjects were excluded due to the following reasons: 1 male met criteria of substance dependence, 1 male and 2 females were on psychoactive medication, 2 males and 3 females did not respond/did not show up on the day of the scan, 1 male and 3 females had a history of neurological or psychiatric disorders, and 1 female had MRI incompatibilities. Finally, 42 participants (22 females and 20 males) were included in the study. Additionally, for resting-state analysis 1 male was excluded due to excessive head motion.

Written informed consent was obtained from all participants after full explanation of procedures. The study was approved by the ethics committee of the Hospital Clinic of Barcelona. Subjects of this study were participants of an ongoing study funded by the Spanish Ministry of Science and Innovation (PSI2014-58004-P).

To exclude the presence of psychiatric disorders, the Mini International Neuropsychiatric Interview (Sheehan et al., 1998) was administered. Empathy was assessed with the Cognitive and Affective Empathy Test (TECA, López Pérez et al., 2008), which provides a global score of empathy and is divided into 4 subscales: 2 assessing cognitive empathy (Perspective Taking and Emotional Understanding) and 2 assessing affective empathy (Empathic Distress and Empathic Happiness).

Briefly, the Perspective Taking scale assesses the intellectual ability of putting oneself in someone else’s place. The Emotional Understanding scale measures the ability of acknowledging and understanding the emotional states, intentions, and impressions of others. Within the affective scales, Emotional Distress is the ability of sharing others’ negative emotions, such as pain (Bernhardt and Singer, 2012). Finally, Empathic Happiness is the ability of sharing others’ positive emotions; in other words, to be happy when something good happens to another person (López Pérez et al., 2008).

Scores were transformed into T scores as recommended in the TECA manual (López Pérez et al., 2008) and two groups were established: T scores ≤55 were considered as low empathy and T scores ≥56 were considered as high empathy.

### MRI Acquisition and Preprocessing

Magnetic resonance images were acquired with a 3T scanner (MAGNETOM Trio, Siemens, Germany), using an 8-channel head coil. The scanning protocol included high-resolution three-dimensional T1-weighted images acquired in the sagittal plane (TR = 2,300 ms, TE = 2.98 ms, TI = 900 ms, 240 slices, FOV = 256 mm; matrix size = 256 × 256; 1 mm isotropic voxel) and a resting-state 10-min-long functional gradient-echo echo-planar imaging sequence (240 T2^∗^ weighted images, TR = 2.5 s, TE = 28 ms, flip angle = 80°, slice thickness = 3 mm, FOV = 240 mm). Subjects were instructed to keep their eyes closed, not to fall asleep, and not to think anything in particular.

#### Cortical Thickness

Cortical thickness was estimated using the automated FreeSurfer stream (version 6.0^1^). The procedures carried out by FreeSurfer include removal of non-brain data, intensity normalization (Fischl et al., 2001), tessellation of the GM / white matter (WM) boundary, automated topology correction (Dale et al., 1999; Ségonne et al., 2007), and accurate surface deformation to identify tissue borders (Dale and Sereno, 1993; Fischl and Dale, 2000; Fischl et al., 2002). Cortical thickness is then calculated as the distance between the WM and GM surfaces at each vertex of the reconstructed cortical mantle (Fischl et al., 2002). After FreeSurfer preprocessing, results for each subject were visually inspected to ensure accuracy of registration, skull stripping, segmentation, and cortical surface reconstruction. Maps were smoothed using a circularly symmetric Gaussian kernel across the surface with a full width at half maximum (FWHM) of 15 mm.

#### Subcortical Volumes

Six subcortical volumes (amygdala, hippocampus, nucleus accumbens, thalamus, caudate, and putamen) and estimated total intracranial volume (eTIV) were obtained via whole-brain segmentation (Fischl et al., 2002). Ratios were calculated for all subcortical structures to eTIV (left or right-hemisphere subcortical structure / eTIV)^∗^100).

#### Resting-State Images

Basic functional image preprocessing, using AFNI^2^ tools, included: discarding the first 5 volumes to allow magnetization stabilization, despiking, motion correction, grand-mean scaling, linear detrending, and temporal filtering (maintaining frequencies above 0.01 Hz).

For connectivity analysis, based on previous literature we defined 6 regions of interest: the bilateral orbitofrontal, cingulate, and insular cortices, amygdala, hippocampus, and thalamus. The corresponding masks were extracted from the Brainnetome Atlas, which is built on functional and anatomical images^3^. We merged all brainnetome subregions corresponding to each selected region (see Supplementary Table 1). Since this atlas is registered to MNI space, (unsmoothed) resting-state images normalized to standard MNI space (voxel size: 3 mm × 3 mm × 3 mm) were used to extract the signal variation time course of the regions of interest.

#### Noise Correction and Head Motion

Regarding head motion parameters, an exclusion cut-off was established for mean interframe head motion at ≥0.3 mm translation or 0.3° rotation; and for maximum interframe head motion at ≥1 mm translation or 1° rotation. As described in the participants section, we excluded 1 male participant due to excessive head movement (maximum rotation: 3.06°).

In order to remove the effects of head motion and other non-neural sources of signal variation from the functional data, we used an Independent Component Analysis (ICA)-based strategy for Automatic Removal of Motion Artifacts (ICA-AROMA, Pruim et al., 2015). ICA-AROMA decomposes the data via ICA and automatically identifies which of these components are related to head motion, by using four robust and standardized features.

As quality control measure to assess the efficacy of ICA-AROMA in reducing relationship between signal variation and motion, we performed correlations between framewise head displacement (Power et al., 2012) and overall signal variation (defined as the voxel-wise root mean square intensity difference between subsequent time points) after regressing the ICA-AROMA components. These two measures should not correlate significantly because signal change should not be explained by head motion. Supplementary Table 2 summarizes groups’ means of all motion parameters.

### Statistical Analysis

#### Demographics and Empathy Scores

Demographic and volumetric statistical analyses were conducted using IBM SPSS Statistics 25.0 (2011; IBM Corp, Armonk, NY, United States). We tested for group differences in demographics between females and males and between groups of high and low empathy for each test scale using the Mann-Whitney *U*-test for non-normally distributed quantitative measures as indicated by the Shapiro-Wilk test; for normally distributed measures, Student’s *T*-test was used. Fisher’s exact test was used for categorical measures.

#### Cortical Thickness Analyses

Intergroup cortical thickness comparisons were performed using a vertex-by-vertex general linear model with FreeSurfer. The model included cortical thickness as a dependent factor and the low/high groups of empathy from each subscale as independent factors. Scores in the Vocabulary subtest from the Wechsler Adults Intelligence Scale-IV (Wechsler, 2008) were entered as a covariate of no interest. All results were corrected for multiple comparisons using pre-cached cluster-wise Monte Carlo simulation with 10,000 iterations. Reported cortical regions reached a two-tailed corrected significance level of *p* < 0.05. Mean thickness (mm) from significant clusters was extracted for plotting results.

#### Subcortical Volumes

Group differences between groups of high and low empathy in subcortical volumes were tested with the Hotelling’s T-squared distribution test for multivariate ANOVA and *F*-test for univariate test stats.

#### Resting-State Analyses

The first eigenvariate of the time series of all voxels included in each of the six masks described above (see Resting-state images section) was extracted with the fslmeants tool^4^. The first eigenvariate represents the weighted mean of the data that results in the time series with maximum possible variance. We then fitted a general linear model with the preprocessed images and the time series extracted. At this step, we used smoothed images (smoothed at full width half maximum of 6) to include them into the general linear model. Nuisance factors from ICA-AROMA, the six head motion parameters extracted during motion correction, and the mean ventricular and WM time series were included as regressors. Finally, six binary masks were created from the five other regions left and we tested for group differences within each TECA scale group using FSL’s randomize permutation-testing tool (5,000 permutations, Winkler et al., 2014). Therefore, for each TECA scale, six permutation testing analyses were performed. To correct for multiple comparisons across voxels we used the threshold-free cluster enhancement (TFCE, Smith and Nichols, 2009) method and significance *p*-value threshold was set at *P* < 0.05 / (2^∗^6) = 0.004 after Bonferroni multiple comparison correction; being 2 the number of contrasts per region of interest and 6 the number of masks used. We also set a cluster-size threshold of 50 voxels in intergroup analyses.

## Results

### Demographics

There were no significant differences in demographical variables between gender groups. Females scored significantly higher than males in all the empathic scales except for the Perspective Taking scale. However, no gender differences were found between groups of low and high empathy (Table 1). Additionally, there were no other demographical differences between groups of high and low empathy for each of the five test scales.

**Table 1:** Demographical and empathy variables.

	Males (*n* = 20)	Females (*n* = 22)	Test stat	*p*-value	Total sample (*n* = 42)
Age, median (IQR)	22.5 (8.0)	19.0 (2.0)	150.500^1^	0.071	19.0 (5.0)
Education, years, median (IQR)	13.5 (6.0)	13.00 (2.0)	184.500^1^	0.353	13.0 (4.0)
Vocabulary test^∗^, median (IQR)	38.0 (8.0)	38.0 (6.0)	224.000^1^	0.714	38.0 (6.0)
TECA total score, mean (SD)	114.2 (12.0)	132.4 (13.4)	4.641^2^	<0.001	123.7 (15.6)
TECA total, low/high (%)	10 (50.0) / 10 (50.0)	6 (27.3) / 16 (72.7)	0.204	0.116	16 (38.1) / 26 (61.9)
Perspective taking, mean (SD)	31.4 (4.5)	33.4 (4.3)	1.492^2^	0.144	32.4 (4.4)
Perspective taking, low/high (%)	8 (40.0) / 12 (60.0)	6 (27.3) / 16 (72.7)	0.515	0.293	14 (33.3) / 28 (66.7)
Emotional understanding, mean (SD)	31.9 (5.7)	35.6 (4.2)	2.440^2^	0.019	33.9 (5.2)
Emotional understanding, low/high (%)	9 (45.0) / 11 (55.0)	7 (31.8) / 15 (68.2)	0.527	0.288	16 (38.1) / 26 (61.9)
Empathic distress, mean (SD)	19.5 (6.5)	28.0 (5.7)	4.494^2^	<0.001	24.0 (7.4)
Empathic distress, low/high (%)	16 (80.0) / 4 (20.0)	13 (59.1) / 9 (40.9)	0.190	0.129	29 (69.0) / 13 (31.0)
Empathic happiness, median (IQR)	32.0 (7.0)	36.5 (7.0)	333.000^1^	0.004	34.0 (7.0)
Empathic happiness, low/high (%)	11 (55.0) / 9 (45.0)	9 (40.9) / 13 (59.1)	0.537	0.273	20 (47.6) / 22 (52.4)


*^1^Mann-Whitney *U*-test; ^*2*^Student’s *t*-test; ^∗^Vocabulary subtest of the Wechsler Adult Intelligence Scale-IV; SD, standard deviation; IQR, interquartile range; TECA, Test of Cognitive and Affective Empathy. Data are shown as mean (SD) for normally distributed quantitative measures; median (IQR) for non-normally distributed measures and as frequency (percentage) for categorical variables. Fisher’s exact test was used for categorical variables. Test stats are group comparisons between males and females.*

### Whole-Brain Cortical Thickness

**FIGURE 1:**
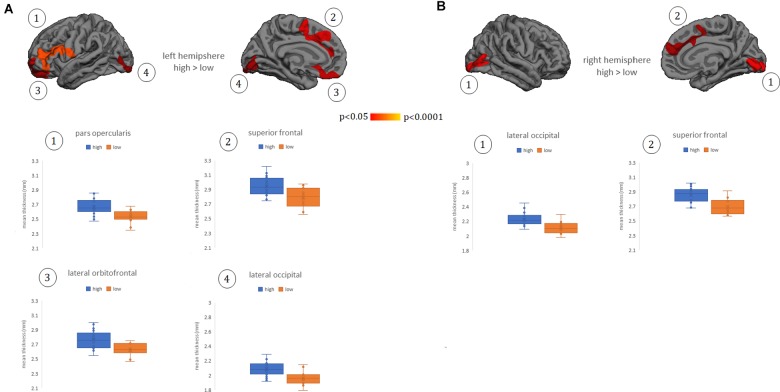
Cortical thickness differences between groups of low and high empathy within the Perspective Taking subscale. **(A)** left hemisphere. **(B)** right hemisphere. Numbers indicate each cluster of significance that in turn are plotted below the cortical maps. Results were corrected using Monte Carlo simulation at two-tailed *p* < 0.05. Color maps indicate significant cortical thickening in the high group compared with the low group. Boxplots show the mean thickness values of each participant within the clusters that reached significant differences between groups. Vocabulary test scores were used as a covariate. The box of the graphs indicates the second and third quartile and middle lines are medians.

Whole-brain cortical thickness comparisons showed that subjects grouped in the high Perspective Taking scores (cognitive empathy) showed thicker cortex in left lateral and medial orbitofrontal gyrus, lateral pars opercularis extending to pars triangularis, and inferior frontal gyrus, as well as in bilateral medial superior frontal, anterior and middle cingulate gyrus, and lateral and medial occipital regions (Figure 1A,B). There were no other significant differences between groups in other subscales or in the TECA global score.

### Subcortical Regions

Subcortical volumetric analyses showed that participants grouped in the high Empathic Distress scale had significantly higher bilateral thalamus volumes than the low-empathy group, although the multivariate test was not statistically significant (Supplementary Table 3).

### Seed-Based Resting-State

For the resting-state permutation testing analyses, no head motion parameters (e.g., framewise displacement, rotation, and translation) were considered as covariates since there were no significant differences between high and low empathy groups.

**FIGURE 2:**
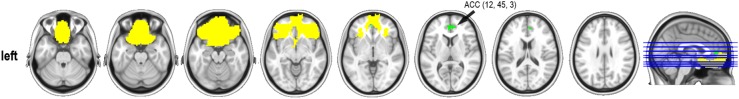
Resting-state connectivity group differences in the Empathic Happiness subscale. ACC, anterior cingulate cortex (MNI coordinates). Represented in yellow, the orbital mask from the Brainnetome atlas and in green the cluster that reached statistical significance at *P*-corrected < 0.004 after Bonferroni multiple comparisons correction. Contrast group was low empathy > high empathy. Cluster-size threshold was set at 50 voxels. Thus, low empathic individuals had stronger functional connectivity between the orbitofrontal cortex and the anterior cingulate gyrus than the high empathic group in the Empathic happiness scale.

Significant differences were found in one of the affective empathy scales, the Empathic Happiness. Subjects classified as having low empathy had increased connectivity between the bilateral orbitofrontal and the anterior cingulate regions (x,y,z MNI coordinates: 12,45,3; 67 voxels in the cluster; max *t*-test = 4.440; *P*-value = 0.003) when compared with the high empathy group (Figure 2). There were no other group differences in resting-state connectivity in any other selected regions at *P*-corrected < 0.004.

## Discussion

The novelty of this study is the characterization of distinct neuroanatomical and functional correlates of cognitive and affective empathy in the same sample of healthy young adults. Overall, our findings showed that the orbitofrontal and cingulate cortices were related to both empathic dimensions. Higher cognitive empathy was associated with orbitofrontal thickening extending to the ventrolateral prefrontal cortex and bilateral superior frontal, cingulate, and occipital cortices. On the other hand, high negative affective empathy was linked to higher bilateral thalamus volumetry. The low positive affective empathy group had higher connectivity at rest between the bilateral orbitofrontal and cingulate cortices.

According to our structural and functional results, the orbitofrontal and anterior cingulate cortices seem to be key structures involved in empathy. However, cortical thickness was better able to discriminate between individuals with high and low empathy than resting state functional connectivity. The fact that the structural neuroanatomical information was more informative than functional connectivity is compatible with the notion that empathy was measured as a personality trait (e.g., “to understand how another person feels is something really easy to me”) rather than a state (Leiberg and Anders, 2006).

Group differences in cortical thickness between groups of high and low empathy within the Perspective Taking subtest were observed in both medial and lateral orbital cortices. Previous structural MRI studies also pointed to the dorsomedial prefrontal cortex as an anatomical substrate of empathy in healthy subjects using cortical thickness measures both studying cortical parcellations (Massey et al., 2017) and from a whole-brain approach (Valk et al., 2016). Earlier studies evidenced dorsomedial prefrontal correlations with empathy using a different methodological approach based on GM density (Eres et al., 2015).

In pathological conditions, cortical thickness correlates in lateral and medial prefrontal cortices have been described. For example, in pathological narcissism, which is characterized by arrogant behavior and lack of empathy, volumetric reductions, and cortical thinning in the right dorsolateral prefrontal cortex have been found (Mao et al., 2016). Also, reduced cortical thickness in inferior, middle, and superior frontal gyri was related to cognitive empathy in individuals at high risk of alcohol abuse (Schmidt et al., 2017). Dysfunctions in social cognition have also been described in neurological disorders (Henry et al., 2016). In a study with patients diagnosed with the behavioral variant of frontotemporal dementia, Perspective Taking scores correlated with atrophy in extensive parts of the right dorsolateral prefrontal cortex (Eslinger et al., 2011). From fMRI task-based studies, Fan et al. (2011) suggest that the left anterior insula is the core of the empathy network while there is regional specificity for both cognitive and affective dimensions, being the left orbitofrontal a cognitive-related region.

Structurally, we also found thicker bilateral cingulate cortex in the group with high perspective-taking empathy than in the low-empathy group. Positive correlations between cognitive empathy and the anterior cingulate (Banissy et al., 2012; Massey et al., 2017) as well as the middle cingulate cortex (Eres et al., 2015) have been reported in healthy individuals. In a study of patients with frontal lesions, it has been found that patients with lesions located in the medial prefrontal cortex extending to the anterior cingulate gyrus had poor cognitive empathic abilities (Shamay-Tsoory et al., 2009).

In the present study, group connectivity differences were found between the bilateral orbitofrontal with the anterior cingulate in the scale evaluating positive affective empathy. Within the limbic system, the cingulate cortex has been reported as a hub region, defined as a region that integrates different brain processes (Van Den Heuvel and Sporns, 2013). Indeed, the rostral anterior cingulate cortex projects to lateral and orbital regions of the prefrontal cortex (Bernhardt and Singer, 2012). Interestingly, we found regional thickening in these regions. Recent psychopathological studies using resting state images, showed orbitofrontal anomalies in conditions characterized by a lack of empathy including individuals with autism (Bi et al., 2018) and psychopathic subjects (Espinoza et al., 2018). In this last study, brain anomalies were also reported in the anterior and posterior cingulate. Our results suggest that subjects with low affective empathy with no previous psychiatric condition would over-engage cognitive-empathic pathways. However, it is important to highlight that in the literature of empathy in autism and psychopathy, there is an ongoing discussion in whether a lack of empathy is genuine or not (Decety et al., 2013; Richman and Bidshahri, 2018).

In our results, we also found regional thickness differences in bilateral occipital cortex involving the pericalcarine and lingual gyri using whole-brain cortical thickness analysis. Since most of the structural studies performed with healthy subjects have focused on regions selected *a priori*, literature relating cognitive empathy to non-limbic brain areas is scant. Similarly to our results, Valk et al. (2016), performing a whole-brain approach, reported the right occipital and fusiform gyri as anatomical substrates of mentalizing. This work is relevant since they combined structural vertex-wise whole brain analyses with an empathic fMRI task. In pathological conditions, Hadjikhani et al. (2006) found cortical thickness reductions in the left inferior occipital region in high-functioning autism spectrum disorders adults, and Schmidt et al. (2017) found reduced cortical thickness in the right precuneus in subjects at high risk of alcoholism and low empathy.

In the current study, analyses of volumetric subcortical structures showed that participants with high Empathic Distress scale scores had significantly higher bilateral thalamus volumes compared with those in the low-empathy group. Previous MRI structural studies have also found a link between the thalamus and both affective and cognitive empathy. One study investigated the neural correlates of both empathy and alexithymia in a sample of healthy participants and reported the thalamus, together with other structures including left amygdala, hippocampus and parahippocampal gyrus as significant correlates of both constructs (Goerlich-Dobre et al., 2015). Similarly, lower affective and cognitive empathy in individuals with temporal lobe epilepsy was associated with smaller fronto-limbic regions including the thalamus (Toller et al., 2015). The thalamus is a complex structure that makes multiple projections to other subcortical structures and the neocortex. The so-called limbic thalamus connects with other limbic structures and has been associated with stress and anxiety states (Vertes et al., 2015).

Global TECA scores did not reveal any significant differences between groups either in cortical or subcortical structures or in functional connectivity, thus emphasizing the importance of differentiating between the cognitive and the affective empathy, with different underlying neural bases. Sex differences were found in all TECA subscales and global scores as previously reported in the literature (Bratek et al., 2015; Esquerda et al., 2016), although these differences disappeared when considering the construct of empathy as binomial (high/low empathy). The actual influence of sex in empathy is still under debate.

Previous studies mainly focused on *a priori* regions already described in the literature from functional MRI studies. One of the strengths and novelty of the present study is the whole-brain vertex-wise methodology used to compare groups in the structural analyses. A second strength is that all participants were similar on demographical variables (i.e., age and education level). Regarding functional connectivity analyses, results reported in the text survived all multiple comparison corrections applied.

The main limitation is the small sample size. Unlike in the whole-brain approach used in the structural analysis, we decided to select *a priori* regions for resting-state connectivity analyses to increase the detection power. Additionally, the study sample was composed of healthy young subjects with no neurological or psychiatric conditions, which makes subtler the neuroanatomical correlates linked to personality traits.

## Conclusion

In conclusion, we found that structural differences between individuals with high and low empathy are more marked than functional ones. Cognitive empathy had clear correlates with cortical structures, namely medial and lateral prefrontal cortices and associative occipital ones. For affective empathy, only a link with the thalamus was observed. However, in the absence of neuroanatomical differences in positive affective empathy, individuals with low empathy showed increased orbital functional connectivity with the anterior cingulate.

## Ethics Statement

The study was approved by the ethics committee of the Hospital Clinic of Barcelona. Subjects of this study were participants of an ongoing study funded by the Spanish Ministry of Science and Innovation (PSI2014-58004-P). Written informed consent was obtained from all participants after full explanation of procedures.

## Author Contributions

CJ contributed in the design of the study. CU, AP-D, and AA contributed to the analysis of the data. CU, AP-D, AA, HB, BS, and CJ contributed to the interpretation of the data, revised the manuscript critically for important intellectual content, and approved the final version of the manuscript. AP-D wrote a first draft. CU and CJ modified the first draft of the article.

## Conflict of Interest Statement

The authors declare that the research was conducted in the absence of any commercial or financial relationships that could be construed as a potential conflict of interest.

## Footnotes

1 https://surfer.nmr.mgh.harvard.edu/

2 https://afni.nimh.nih.gov/afni

3 https://atlas.brainnetome.org/bnatlas.html

4 https://fsl.fmrib.ox.ac.uk/fsl/fslwiki/Fslutils

## References

[B1] Banissy M. J. Kanai R. Walsh V. Rees G. (2012). Inter-individual differences in empathy are reflected in human brain structure. *Neuroimage* 62 2034–2039. 10.1016/j.neuroimage.2012.05.081 22683384 PMC3778747

[B2] Bernhardt B. C. Singer T. (2012). The neural basis of empathy. *Annu. Rev. Neurosci.* 35 1–23. 10.1146/annurev-neuro-062111-150536 22715878

[B3] Bi X.-A. Wang Y. Shu Q. Sun Q. Xu Q. (2018). Classification of autism spectrum disorder using random support vector machine cluster. *Front. Genet.* 9:18 10.3389/fgene.2018.00018 PMC5808191 29467790

[B4] Bilevicius E. Kolesar T. A. Smith S. D. Trapnell P. D. Kornelsen J. (2018). Trait emotional empathy and resting state functional connectivity in default mode, salience, and central executive networks. *Brain Sci.* 8 1–11. 10.3390/brainsci8070128 29986390 PMC6071260

[B5] Braadbaart L. de Grauw H. Perrett D. I. Waiter G. D. Williams J. H. G. (2014). The shared neural basis of empathy and facial imitation accuracy. *Neuroimage* 84 367–375. 10.1016/j.neuroimage.2013.08.061 24012546

[B6] Bratek A. Bulska W. Bonk M. Seweryn M. Krysta K. (2015). Empathy among physicians, medical students and candidates. *Psychiatr. Danub.* 27 S48–S52. 26417736

[B7] Dale A. M. Fischl B. Sereno M. I. (1999). Cortical surface-based analysis: I. Segmentation and surface reconstruction. *Neuroimage* 9 179–194. 10.1006/nimg.1998.0395 9931268

[B8] Dale A. M. Sereno M. I. (1993). Improved localizadon of cortical activity by combining EEG and MEG with MRI cortical surface reconstruction: a linear approach. *J. Cogn. Neurosci.* 5 162–176. 10.1162/jocn.1993.5.2.162 23972151

[B9] Decety J. Chen C. Harenski C. Kiehl K. A. (2013). An fMRI study of affective perspective taking in individuals with psychopathy: imagining another in pain does not evoke empathy. *Front. Hum. Neurosci.* 7:489. 10.3389/fnhum.2013.00489 24093010 PMC3782696

[B10] Decety J. Norman G. J. Berntson G. G. Cacioppo J. T. (2012). A neurobehavioral evolutionary perspective on the mechanisms underlying empathy. *Prog. Neurobiol.* 98 38–48. 10.1016/j.pneurobio.2012.05.001 22580447

[B11] Dvash J. Shamay-Tsoory S. G. (2014). Theory of mind and empathy as multidimensional constructs: neurological foundations. *Top. Lang. Disord.* 34 282–295. 10.1097/TLD.0000000000000040

[B12] Eres R. Decety J. Louis W. R. Molenberghs P. (2015). Individual differences in local gray matter density are associated with differences in affective and cognitive empathy. *Neuroimage* 117 305–310. 10.1016/j.neuroimage.2015.05.038 26008886

[B13] Eslinger P. J. Moore P. Anderson C. Grossman M. (2011). Social cognition, executive functioning, and neuroimaging correlates of empathic deficits in frontotemporal dementia. *J. Neuropsychiatry Clin. Neurosci.* 23 74–82. 10.1176/jnp.23.1.jnp74 21304142 PMC3641646

[B14] Espinoza F. A. Vergara V. M. Reyes D. Anderson N. E. Harenski C. L. Decety J. (2018). Aberrant functional network connectivity in psychopathy from a large (N = 985) forensic sample. *Hum. Brain Mapp.* 39 2624–2634. 10.1002/hbm.24028 29498761 PMC5951759

[B15] Esquerda M. Yuguero O. Viñas J. Pifarré J. (2016). La empatía médica, >nace o se hace? Evolución de la empatía en estudiantes de medicina. *Atención Primaria* 48 8–14. 10.1016/j.aprim.2014.12.012 26027760 PMC6880110

[B16] Fan Y. Duncan N. W. de Greck M. Northoff G. (2011). Is there a core neural network in empathy? An fMRI based quantitative meta-analysis. *Neurosci. Biobehav. Rev.* 35 903–911. 10.1016/j.neubiorev.2010.10.009 20974173

[B17] Fischl B. Dale A. M. (2000). Measuring the thickness of the human cerebral cortex from magnetic resonance images. *Proc. Natl. Acad. Sci. U.S.A.* 97 11050–11055. 10.1073/pnas.200033797 10984517 PMC27146

[B18] Fischl B. Liu A. Dale A. M. (2001). Automated manifold surgery: constructing geometrically accurate and topologically correct models of the human cerebral cortex. *IEEE Trans. Med. Imaging* 20 70–80. 10.1109/42.906426 11293693

[B19] Fischl B. Salat D. H. Busa E. Albert M. Dieterich M. Haselgrove C. (2002). Whole brain segmentation: automated labeling of neuroanatomical structures in the human brain. *Neuron* 33 341–355. 11832223 10.1016/s0896-6273(02)00569-x

[B20] Goerlich-Dobre K. S. Lamm C. Pripfl J. Habel U. Votinov M. (2015). The left amygdala: a shared substrate of alexithymia and empathy. *Neuroimage* 122 20–32. 10.1016/j.neuroimage.2015.08.014 26275382

[B21] Gonzalez-Liencres C. Shamay-Tsoory S. G. Brüne M. (2013). Towards a neuroscience of empathy: ontogeny, phylogeny, brain mechanisms, context and psychopathology. *Neurosci. Biobehav. Rev.* 37 1537–1548. 10.1016/j.neubiorev.2013.05.001 23680700

[B22] Hadjikhani N. Joseph R. M. Snyder J. Tager-Flusberg H. (2006). Anatomical differences in the mirror neuron system and social cognition network in autism. *Cereb. Cortex* 16 1276–1282. 10.1093/cercor/bhj069 16306324

[B23] Harvey P.-O. Zaki J. Lee J. Ochsner K. Green M. F. (2013). Neural substrates of empathic accuracy in people with schizophrenia. *Schizophr. Bull.* 39 617–628. 10.1093/schbul/sbs042 22451493 PMC3627780

[B24] Henry J. D. Von Hippel W. Molenberghs P. Lee T. Sachdev P. S. (2016). Clinical assessment of social cognitive function in neurological disorders. *Nat. Rev. Neurol.* 12 28–39. 10.1038/nrneurol.2015.229 26670297

[B25] Lamm C. Batson C. D. Decety J. (2007). The neural substrate of human empathy: effects of perspective-taking and cognitive appraisal. *J. Cogn. Neurosci.* 19 42–58. 10.1162/jocn.2007.19.1.42 17214562

[B26] Lamm C. Decety J. Singer T. (2011). Meta-analytic evidence for common and distinct neural networks associated with directly experienced pain and empathy for pain. *Neuroimage* 54 2492–2502. 10.1016/j.neuroimage.2010.10.014 20946964

[B27] Leiberg S. Anders S. (2006). The multiple facets of empathy: a survey of theory and evidence. *Prog. Brain Res.* 156 419–440. 10.1016/S0079-6123(06)56023-6 17015094

[B28] López Pérez B. Fernández Pinto I. Abad García F. J. (2008). *Test de Empatía Cognitiva y Afectiva*. Madrid: TEA.

[B29] Mao Y. Sang N. Wang Y. Hou X. Huang H. Wei D. (2016). Reduced frontal cortex thickness and cortical volume associated with pathological narcissism. *Neuroscience* 328 50–57. 10.1016/j.neuroscience.2016.04.025 27129440

[B30] Massey S. H. Stern D. Alden E. C. Petersen J. E. Cobia D. J. Wang L. (2017). Cortical thickness of neural substrates supporting cognitive empathy in individuals with schizophrenia. *Schizophr. Res.* 179 119–124. 10.1016/j.schres.2016.09.025 27665257 PMC5222696

[B31] Moore R. C. Dev S. I. Jeste D. V. Dziobek I. Eyler L. T. (2015). Distinct neural correlates of emotional and cognitive empathy in older adults. *Psychiatry Res. Neuroimaging* 232 42–50. 10.1016/j.pscychresns.2014.10.016 25770039 PMC4404184

[B32] Mutschler I. Reinbold C. Wankerl J. Seifritz E. Ball T. (2013). Structural basis of empathy and the domain general region in the anterior insular cortex. *Front. Hum. Neurosci.* 7:177. 10.3389/fnhum.2013.00177 23675334 PMC3648769

[B33] Power J. D. Barnes K. A. Snyder A. Z. Schlaggar B. L. Petersen S. E. (2012). Spurious but systematic correlations in functional connectivity MRI networks arise from subject motion. *Neuroimage* 59 2142–2154. 10.1016/j.neuroimage.2011.10.018 22019881 PMC3254728

[B34] Pruim R. H. R. Mennes M. van Rooij D. Llera A. Buitelaar J. K. Beckmann C. F. (2015). ICA-AROMA: a robust ICA-based strategy for removing motion artifacts from fMRI data. *Neuroimage* 112 267–277. 10.1016/j.neuroimage.2015.02.064 25770991

[B35] Richman K. A. Bidshahri R. (2018). Autism, theory of mind, and the reactive attitudes. *Bioethics* 32 43–49. 10.1111/bioe.12370 28914977

[B36] Schmidt T. Roser P. Ze O. Juckel G. Suchan B. Thoma P. (2017). Cortical thickness and trait empathy in patients and people at high risk for alcohol use disorders. *Psychopharmacology* 234 3521–3533. 10.1007/s00213-017-4741-3 28971228

[B37] Ségonne F. Pacheco J. Fischl B. (2007). Geometrically accurate topology-correction of cortical surfaces using nonseparating loops. *IEEE Trans. Med. Imaging* 26 518–529. 10.1109/TMI.2006.887364 17427739

[B38] Shamay-Tsoory S. G. Aharon-Peretz J. Perry D. (2009). Two systems for empathy: a double dissociation between emotional and cognitive empathy in inferior frontal gyrus versus ventromedial prefrontal lesions. *Brain* 132 617–627. 10.1093/brain/awn279 18971202

[B39] Sheehan D. V. Lecrubier Y. Sheehan K. H. Amorim P. Janavs J. Weiller E. (1998). The mini-international neuropsychiatric interview (M.I.N.I.): the development and validation of a structured diagnostic psychiatric interview for DSM-IV and ICD-10. *J. Clin. Psychiatry* 59(Suppl. 20), 22–33. 10.1016/S0924-9338(99)80239-9 9881538

[B40] Smith S. M. Nichols T. E. (2009). Threshold-free cluster enhancement: addressing problems of smoothing, threshold dependence and localisation in cluster inference. *Neuroimage* 44 83–98. 10.1016/J.NEUROIMAGE.2008.03.061 18501637

[B41] Takeuchi H. Taki Y. Nouchi R. Sekiguchi A. Hashizume H. Sassa Y. (2014). Association between resting-state functional connectivity and empathizing/systemizing. *Neuroimage* 99 312–322. 10.1016/j.neuroimage.2014.05.031 24844739

[B42] Takeuchi H. Taki Y. Nouchi R. Sekiguchi A. Hashizume H. Sassa Y. (2018). The neural substrate of human empathy: effects of perspective-taking and cognitive appraisal. *Neuroimage* 8 312–322. 10.1162/jocn.2007.19.1.42 17214562

[B43] Toller G. Adhimoolam B. Rankin K. P. Huppertz H.-J. Kurthen M. Jokeit H. (2015). Right fronto-limbic atrophy is associated with reduced empathy in refractory unilateral mesial temporal lobe epilepsy. *Neuropsychologia* 78 80–87. 10.1016/j.neuropsychologia.2015.09.010 26363299

[B44] Valk S. L. Bernhardt B. C. Böckler A. Trautwein F. M. Kanske P. Singer T. (2016). Socio-cognitive phenotypes differentially modulate large-scale structural covariance networks. *Cereb. Cortex* 27 1358–1368. 10.1093/cercor/bhv319 26733538

[B45] Van Den Heuvel M. P. Sporns O. (2013). Special issue: the connectome-feature review network hubs in the human brain. *Trends Cogn. Sci.* 17 683–696. 10.1016/j.tics.2013.09.012 24231140

[B46] Van Overwalle F. Baetens K. (2009). Understanding others’ actions and goals by mirror and mentalizing systems: a meta-analysis. *Neuroimage* 48 564–584. 10.1016/j.neuroimage.2009.06.009 19524046

[B47] Vertes R. P. Linley S. B. Hoover W. B. (2015). Limbic circuitry of the midline thalamus. *Neurosci. Biobehav. Rev.* 54 89–107. 10.1016/J.NEUBIOREV.2015.01.014 25616182 PMC4976455

[B48] Walter H. (2012). Social cognitive neuroscience of empathy: concepts, circuits, and genes. *Emot. Rev.* 4 9–17. 10.1177/1754073911421379

[B49] Wechsler D. (2008). *Wechsler Adult Intelligence Test*, Fourth Edn San Antonio, TX: Psychological Corporation.

[B50] Winkler A. M. Ridgway G. R. Webster M. A. Smith S. M. Nichols T. E. (2014). Permutation inference for the general linear model. *Neuroimage* 92 381–397. 10.1016/j.neuroimage.2014.01.060 24530839 PMC4010955

