# Supplementary material for: Neuroanatomical and Functional Correlates of Cognitive and Affective Empathy in Young Healthy Adults
Source: Front Behav Neurosci. 2019 May 1;13:85. doi: 10.3389/fnbeh.2019.00085 (PMC6504763; doi:10.3389/fnbeh.2019.00085)
Supplement: Supplementary file 1 [file Data_Sheet_1.docx]

Supplementary Material

**Neuroanatomical and functional correlates of cognitive and affective empathy in young healthy adults**

Carme Uribe^1 #^, Arnau Puig-Davi^1 #^, Alexandra Abos^1^, Hugo C Baggio^1^, Carme Junqué ^1,2,3 *^ and Barbara Segura^1,2^.

^1^Medical Psychology Unit, Department of Medicine. Institute of Neuroscience, University of Barcelona. Barcelona, Catalonia.

^2^Centro de Investigación Biomédica en Red sobre Enfermedades Neurodegenerativas (CIBERNED), Hospital Clínic de Barcelona. Barcelona, Spain.

^3^Institute of Biomedical Research August Pi i Sunyer (IDIBAPS). Barcelona, Catalonia.

**^#^ CU and APD contributed equally to the manuscript.**

*** Correspondence:**Prof. Carme Junque

Medical Psychology Unit, Department of Medicine. University of Barcelona

Casanova 143 (08036) Barcelona, Spain

Phone: (+34) 93 402 45 70 // Fax: (+34) 93 403 52 94 // E-mail: [cjunque@ub.edu](mailto:cjunque@ub.edu)

# Supplementary Tables

## Supplementary Table 1 Regions of the Brainnetome atlas selected for the masks

| ***MNI coordinates***  ***x y z*** |
| --- |
| -7 54 -7 left orbital 6_1 |
| 6 47 -7 right orbital 6_1 |
| -37 33 -16 left orbital 6_2 |
| 40 39 -14 right orbital 6_2 |
| -23 38 -18 left orbital 6_3 |
| 24 36 -18 right orbital 6_3 |
| -6 52 -20 left orbital 6_4 |
| 6 57 -16 right orbital 6_4 |
| -10 18 -19 left orbital 6_5 |
| 9 20 -19 right orbital 6_5 |
| -41 32 -9 left orbital 6_6 |
| 42 31 -9 right orbital 6_6 |
| -36 -20 10 left insula 6_1 |
| 37 -18 8 right insula 6_1 |
| -32 14 -13 left insula 6_2 |
| 33 14 -13 right insula 6_2 |
| -34 18 1 left insula 6_3 |
| 37 19 1 right insula 6_3 |
| -38 -4 -9 left insula 6_4 |
| 39 -2 -9 right insula 6_4 |
| -39 -8 8 left insula 6_5 |
| 39 -7 8 right insula 6_5 |
| -38 5 4 left insula 6_6 |
| 38 5 5 right insula 6_6 |
| -4 -39 31 cingulate gyrus left 7_1 |
| 5 -37 32 cingulate gyrus right 7_1 |
| -3 8 26 cingulate gyrus left 7_2 |
| 5 21 13 cingulate gyrus right 7_2 |
| -6 34 21 cingulate gyrus left 7_3 |
| 5 28 27 cingulate gyrus right 7_3 |
| -8 -47 9 cingulate gyrus left 7_4 |
| 8 -44 11 cingulate gyrus right 7_4 |
| -5 7 37 cingulate gyrus left 7_5 |
| 4 6 38 cingulate gyrus right 7_5 |
| -8 -23 41 cingulate gyrus left 7_6 |
| 6 -20 41 cingulate gyrus right 7_6 |
| -4 39 -2 cingulate gyrus left 7_7 |
| 5 41 7 cingulate gyrus right 7_7 |
| -19 -2 -20 left amygdala 2_1 |
| 19 -3 -20 right amygdala 2_1 |
| -27 -4 -20 left amygdala 2_2 |
| 28 -3 -20 right amygdala 2_2 |
| -22 -14 -19 left hippocampus 2_1 |
| 22 -12 -20 right hippocampus 2_1 |
| -28 -30 -10 left hippocampus 2_2 |
| 29 -27 -10 right hippocampus 2_2 |
| -7 -12 5 left thalamus 8_1 |
| 7 -11 6 right thalamus 8_1 |
| -19 -13 3 left thalamus 8_2 |
| 13 -14 1 right thalamus 8_2 |
| -18 -23 3 left thalamus 8_3 |
| 18 -22 3 right thalamus 8_3 |
| -6 -13 7 left thalamus 8_4 |
| 2 -12 5 right thalamus 8_4 |
| -16 -24 6 left thalamus 8_5 |
| 15 -26 6 right thalamus 8_5 |
| -15 -29 4 left thalamus 8_6 |
| 13 -27 8 right thalamus 8_6 |
| -11 -21 13 left thalamus 8_7 |
| 9 -14 14 right thalamus 8_7 |
| -11 -14 2 left thalamus 8_8 |
| 13 -16 6 right thalamus 8_8 |

## Supplementary Table 2 Motion parameters

|  | **males (n=19)** | **females (n=22)** | **total**  **(n=41)** | **U test (Mann-Whitney)** | **p-value** |
| --- | --- | --- | --- | --- | --- |
| **mean FD, median (IQR)** | 0.12 (0.05) | 0.09 (0.09) | 0.10 (0.08) | 153.0 | 0.143 |
| **maximum FD, median (IQR)** | 0.45 (0.31) | 0.37 (0.29) | 0.39 (0.31) | 159.0 | 0.191 |
| **mean rotation mean, median (IQR)** | 0.03 (0.02) | 0.02 (0.01) | 0.03 (0.01) | 160.0 | 0.200 |
| **maximum rotation, median (IQR)** | 0.17 (0.13) | 0.12 (0.15) | 0.14 (0.14) | 155.0 | 0.158 |
| **mean translation mean, median (IQR)** | 0.06 (0.04) | 0.04 (0.05) | 0.05 (0.04) | 160.0 | 0.200 |
| **maximum translation, median (IQR)** | 0.22 (0.12) | 0.18 (0.17) | 0.21 (0.13) | 161.0 | 0.210 |
| **correlation FD - DVARS, median (IQR)** | 0.05 (0.20) | -0.08 (0.17) | -0.04 (0.21) | 140.0 | 0.071 |

Abbreviations: DVARS, temporal derivative of timecourses of root mean square variance over voxels; FD, framewise displacement; IQR, interquartile range.

## Supplementary Table 3 Limbic subcortical volumes

|  | **high empathy** | **low empathy** | **Univariate F-test** | **p-value** |
| --- | --- | --- | --- | --- |
| ***Perspective Taking* (high = 28; low = 14); t^2^ = 0.461; P = 0.921** | | | | |
| **Left Amygdala** | 0.12 (0.01) | 0.12 (0.01) | 0.144 | 0.707 |
| **Right Amygdala** | 0.12 (0.01) | 0.12 (0.01) | 0.158 | 0.693 |
| **Left Hippocampus** | 0.23 (0.01) | 0.23 (0.01) | 0.198 | 0.658 |
| **Right Hippocampus** | 0.23 (0.01) | 0.23 (0.02) | 0.021 | 0.884 |
| **Left Accumbens** | 0.03 (0.01) | 0.03 (0.00) | 0.214 | 0.646 |
| **Right Accumbens** | 0.04 (0.01) | 0.04 (0.00) | 0.212 | 0.648 |
| **Left Thalamus** | 0.42 (0.03) | 0.41 (0.04) | 0.078 | 0.781 |
| **Right Thalamus** | 0.43 (0.24) | 0.43 (0.04) | 0.019 | 0.890 |
| **Left Putamen** | 0.32 (0.04) | 0.33 (0.03) | 0.396 | 0.533 |
| **Right Putamen** | 0.33 (0.04) | 0.33 (0.03) | 0.000 | 0.998 |
| **Left Caudate** | 0.24 (0.02) | 0.24 (0.02) | 0.469 | 0.497 |
| **Right Caudate** | 0.24 (0.02) | 0.25 (0.02) | 0.883 | 0.353 |
| ***Emotional Understanding* (high = 26; low = 16); t^2^ = 1.107; P = 0.391** | | | | |
| **Left Amygdala** | 0.12 (0.01) | 0.12 (0.01) | 0.146 | 0.704 |
| **Right Amygdala** | 0.12 (0.01) | 0.12 (0.01) | 0.017 | 0.897 |
| **Left Hippocampus** | 0.23 (0.01) | 0.23 (0.01) | 0.075 | 0.785 |
| **Right Hippocampus** | 0.23 (0.01) | 0.23 (0.02) | 0.005 | 0.942 |
| **Left Accumbens** | 0.03 (0.01) | 0.03 (0.00) | 1.978 | 0.167 |
| **Right Accumbens** | 0.04 (0.01) | 0.04 (0.00) | 0.076 | 0.784 |
| **Left Thalamus** | 0.42 (0.29) | 0.41 (0.03) | 0.164 | 0.688 |
| **Right Thalamus** | 0.43 (0.26) | 0.43 (0.03) | 0.151 | 0.700 |
| **Left Putamen** | 0.32 (0.04) | 0.32 (0.03) | 0.120 | 0.731 |
| **Right Putamen** | 0.33 (0.03) | 0.32 (0.03) | 1.257 | 0.269 |
| **Left Caudate** | 00.24 (0.03) | 0.24 (0.02) | 0.033 | 0.857 |
| **Right Caudate** | 0.24 (0.02) | 0.24 (0.02) | 0.006 | 0.939 |
| ***Empathic Distress* (high = 13; low = 29); t^2^ = 0.978; P = 0.491** | | | | |
| **Left Amygdala** | 0.12 (0.01) | 0.12 (0.01) | 0.563 | 0.457 |
| **Right Amygdala** | 0.12 (0.01) | 0.12 (0.01) | 0.538 | 0.468 |
| **Left Hippocampus** | 0.23 (0.01) | 0.23 (0.01) | 0.038 | 0.847 |
| **Right Hippocampus** | 0.23 (0.02) | 0.23 (0.01) | 0.329 | 0.570 |
| **Left Accumbens** | 0.03 (0.00) | 0.03 (0.01) | 0.104 | 0.749 |
| **Right Accumbens** | 0.04 (0.00) | 0.04 (0.01) | 0.135 | 0.715 |
| **Left Thalamus** | 0.43 (0.03) | 0.41 (0.03) | 4.636 | 0.037 |
| **Right Thalamus** | 0.45 (0.03) | 0.42 (0.03) | 6.741 | 0.013 |
| **Left Putamen** | 0.32 (0.04) | 0.32 (0.03) | 0.059 | 0.810 |
| **Right Putamen** | 0.33 (0.04) | 0.33 (0.03) | 0.067 | 0.798 |
| **Left Caudate** | 0.24 (0.02) | 0.24 (0.03) | 0.157 | 0.694 |
| **Right Caudate** | 0.24 (0.02) | 0.24 (0.02) | 0.056 | 0.814 |
| ***Emphatic Happiness* (high = 22; low = 20); t^2^ = 0.442; P = 0.932** | | | | |
| **Left Amygdala** | 0.12 (0.01) | 0.12 (0.01) | 0.227 | 0.636 |
| **Right Amygdala** | 0.12 (0.01) | 0.12 (0.01) | 0.013 | 0.912 |
| **Left Hippocampus** | 0.23 (0.01) | 0.22 (0.02) | 1.461 | 0.234 |
| **Right Hippocampus** | 0.23 (0.01) | 0.23 (0.02) | 0.149 | 0.702 |
| **Left Accumbens** | 0.03 (0.00) | 0.03 (0.00) | 0.841 | 0.365 |
| **Right Accumbens** | 0.04 (0.01) | 0.04 (0.00) | 2.961 | 0.093 |
| **Left Thalamus** | 0.42 (0.03) | 0.41 (0.03) | 1.617 | 0.211 |
| **Right Thalamus** | 0.43 (0.03) | 0.43 (0.03) | 1.234 | 0.273 |
| **Left Putamen** | 0.32 (0.04) | 0.32 (0.03) | 0.178 | 0.675 |
| **Right Putamen** | 0.33 (0.04) | 0.33 (0.03) | 0.345 | 0.560 |
| **Left Caudate** | 0.24 (0.02) | 0.24 (0.03) | 0.001 | 0.975 |
| **Right Caudate** | 0.24 (0.02) | 0.24 (0.02) | 0.004 | 0.948 |
| ***TECA global score* (high = 26; low = 16); t^2^ = 0.491; P = 0.903** | | | | |
| **Left Amygdala** | 0.12 (0.01) | 0.12 (0.01) | 0.005 | 0.947 |
| **Right Amygdala** | 0.12 (0.01) | 0.12 (0.01) | 0.267 | 0.608 |
| **Left Hippocampus** | 0.23 (0.01) | 0.23 (0.01) | 0.196 | 0.661 |
| **Right Hippocampus** | 0.23 (0.01) | 0.23 (0.02) | 0.376 | 0.543 |
| **Left Accumbens** | 0.03 (0.00) | 0.03 (0.00) | 0.001 | 0.974 |
| **Right Accumbens** | 0.04 (0.01) | 0.04 (0.00) | 0.108 | 0.744 |
| **Left Thalamus** | 0.42 (0.03) | 0.41 (0.03) | 1.650 | 0.206 |
| **Right Thalamus** | 0.43 (0.03) | 0.42 (0.03) | 1.916 | 0.174 |
| **Left Putamen** | 0.32 (0.04) | 0.33 (0.03) | 0.582 | 0.450 |
| **Right Putamen** | 0.33 (0.04) | 0.33 (0.02) | 0.353 | 0.555 |
| **Left Caudate** | 0.24 (0.03) | 0.24 (0.02) | 0.399 | 0.531 |
| **Right Caudate** | 0.24 (0.02) | 0.24 (0.02) | 0.278 | 0.601 |

TECA, Test of Cognitive and Affective Empathy.

Data are ratios to estimated intracranial volume and they are shown as means (SD). Multivariate ANOVA test stat is Hotelling’s T-squared distribution statistic. Univariate statistic is F-test.
